# Measures of Anxiety in Zebrafish (*Danio rerio*): Dissociation of Black/White Preference and Novel Tank Test

**DOI:** 10.1371/journal.pone.0036931

**Published:** 2012-05-17

**Authors:** Rachel E. Blaser, Denis B. Rosemberg

**Affiliations:** 1 Department of Psychological Sciences, University of San Diego, San Diego, California, United States of America; 2 Departamento de Bioquímica, Universidade Federal do Rio Grande do Sul, Porto Alegre, Rio Grande do Sul, Brazil; 3 Instituto Nacional de Ciência e Tecnologia em Excitotoxicidade e Neuroproteção (INCT-EN), Porto Alegre, Rio Grande do Sul, Brazil; Cajal Institute, Consejo Superior de Investigaciones Científicas, Spain

## Abstract

The effects of wall color stimuli on diving, and the effects of depth stimuli on scototaxis, were assessed in zebrafish. Three groups of fish were confined to a black, a white, or a transparent tank, and tested for depth preference. Two groups of fish were confined to a deep or a shallow tank, and tested for black-white preference. As predicted, fish preferred the deep half of a split-tank over the shallow half, and preferred the black half of a black/white tank over the white half. Results indicated that the tank wall color significantly affected depth preference, with the transparent tank producing the strongest depth preference and the black tank producing the weakest preference. Tank depth, however, did not significantly affect color preference. Additionally, wall color significantly affected shuttling and immobility, while depth significantly affected shuttling and thigmotaxis. These results are consistent with previous indications that the diving response and scototaxis may reflect dissociable mechanisms of behavior. We conclude that the two tests are complementary rather than interchangeable, and that further research on the motivational systems underlying behavior in each of the two tests is needed.

## Introduction

As the study of zebrafish behavior gains popularity, simple tests have emerged as potentially useful behavioral measures of anxiety. One is the novel tank diving test, which exploits the natural tendency of zebrafish to initially dive to the bottom of a novel experimental tank, with a gradual increase in vertical activity over time [1], [2]. This initial preference for the bottom of the novel tank has been compared to thigmotaxis in rodents [1], [3], and the degree of ‘bottom dwelling’ has been interpreted as an index of anxiety. The black/white preference test exploits another natural tendency of zebrafish, the preference for a black chamber over a white chamber in an experimental tank, which has been suggested to serve a cryptic function [4], [5]. Although both tests have been used to measure ‘anxiety’ in zebrafish [4], [6], [7], [8], [9], the validity of these measures is still under investigation. The convergence of two tests used to measure the same construct provides one form of validation (convergent validity), while divergence between tests suggests that they may not measure the same construct. In order to effectively use these measures to screen for drugs or phenotypes that may affect motivation and behavior, it would be useful to know whether the two tests can be used interchangeably to measure the construct of ‘anxiety’ (chosen as a matter of convenience), or whether they measure dissociable mechanisms of behavior, and if so, how they differ [10].

There may be a useful distinction between stimuli that produce defensive behavior in zebrafish (aversive or fear-inducing stimuli), and those that are utilized in the defensive behaviors. Previous experiments with zebrafish have attempted to use threat cues (social isolation, novel environment), or predator stimuli, such as visual cues (2D or 3D predator models) and olfactory cues (alarm pheromone, water from predator tank) to produce defensive behavior [2], [11], [12], [13], [14], [15], [16]. The behavioral response to these stimuli (for example, avoidance, escape, or immobility) can provide one measure of fear or anxiety. An interesting problem arises, however, when the defensive behaviors are directed at a stimulus other than the ‘causal’ stimulus. Three behaviors commonly measured in zebrafish – scototaxis, thigmotaxis, and the diving response – are defined in relation to a dark location, tank walls, or the tank bottom, respectively. In these cases, it is unclear whether the stimuli themselves have aversive qualities (e.g. whether white or the surface is aversive), or whether the approach/avoidance response is contingent on a motivational state produced by some other aversive stimulus (e.g. isolation, handling, predator stimuli). For example, preference for a cryptic background might be conditional on whether an animal is searching for a mate (low preference) or avoiding a predator (high preference). Such stimuli might be utilized by the animal in a sort of compensatory response, to alleviate the fear induced by a ‘causal’ stimulus. For example, the scent of a predator (causal stimulus) might induce fear, which in turn motivates the animal to approach a shelter (conditional stimulus), which then reduces the state of fear [17]. It could well be that ‘causal’ stimuli (those that induce fear/anxiety) and ‘conditional’ stimuli (those that are approached/avoided conditionally on a state of fear/anxiety and may counteract it) affect distinct mechanisms and could produce unique behavioral profiles.

**Figure 1:**
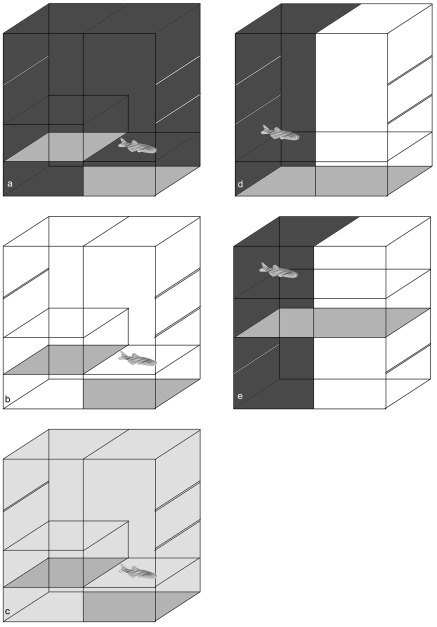
Illustration of the apparatus. In **panels a–c** are the configurations used for examining the effect of color (black in **panel a**, white in **panel b**, transparent in **panel c**) on depth preference. In **panels d and e** are the configurations used for examining the effect of depth (deep in **panel d**, shallow in **panel e**) on color preference. Horizontal open areas represent the plexiglas partitions, while areas filled with grey represent the gravel substrate.

The stimuli employed in the novel tank and the black/white tests could potentially be either causal or conditional. In both cases, avoidance of one stimulus (white compartment, water surface) is observed relative to approach to another stimulus (black compartment, tank bottom). It has not been determined whether the avoided stimuli are intrinsically aversive to fish (causal), or whether they are only avoided within the context of a particular motivational state (conditional). Because virtually all current behavioral tests with zebrafish are likely to induce some fear through handling, tank novelty, and in many instances isolation, animals are seldom tested in a truly neutral or fully habituated state, making it difficult to determine whether these preferences are conditional on fear or anxiety. Even so, there are some predictions that might be used to dissociate ‘causal’ stimuli from ‘conditional’ stimuli in anxiety testing.

**Figure 2:**
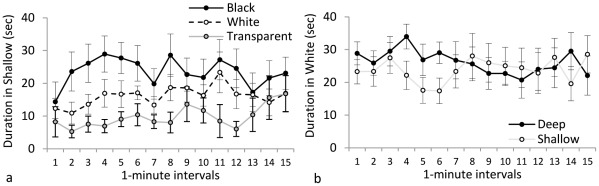
Duration of zebrafish on the less-preferred side. The effect of black, white, and transparent stimuli on depth preference is plotted in **panel a**; animals in black tanks spent more time in the shallow side than those in transparent tanks. The effect of deep and shallow stimuli on color preference is plotted in **panel b**; there was no effect of depth on color preference.

**Figure 3:**
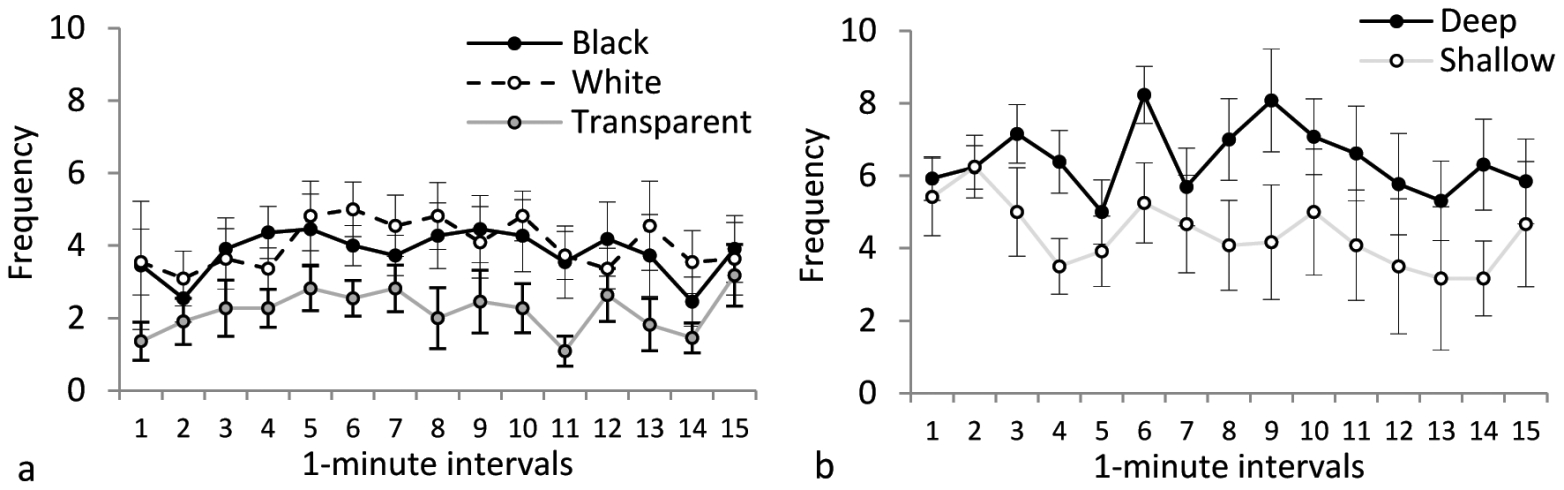
Frequency of shuttling (center-crossing). The effect of black, white, and transparent stimuli on shuttling is plotted in **panel a**; animals in transparent tanks shuttled less frequently than those in black or white tanks. The effect of deep and shallow stimuli on color preference is plotted in **panel b**; animals in shallow tanks shuttled less frequently than those in deep tanks.

First, it might be expected that conditional stimuli will show enhanced habituation relative to causal stimuli. If the white compartment is not actually aversive, but is only avoided when the animal is already afraid, the preference should disappear as fear is reduced. In contrast, if a white compartment is actually aversive, avoidance might continue indefinitely, producing little habituation or even sensitization with repeated exposure [18]. Second, forced exposure to causal stimuli should produce changes in behavior toward conditional stimuli. In contrast, forced exposure to conditional stimuli, having no additive effect on fear, would not be expected to produce changes in behavior toward causal stimuli. Third, it might be predicted that responses to causal stimuli will be less variable than responses to conditional stimuli, since the latter are contingent on the effects of external, and therefore potentially less controlled, stimuli.

**Figure 4:**
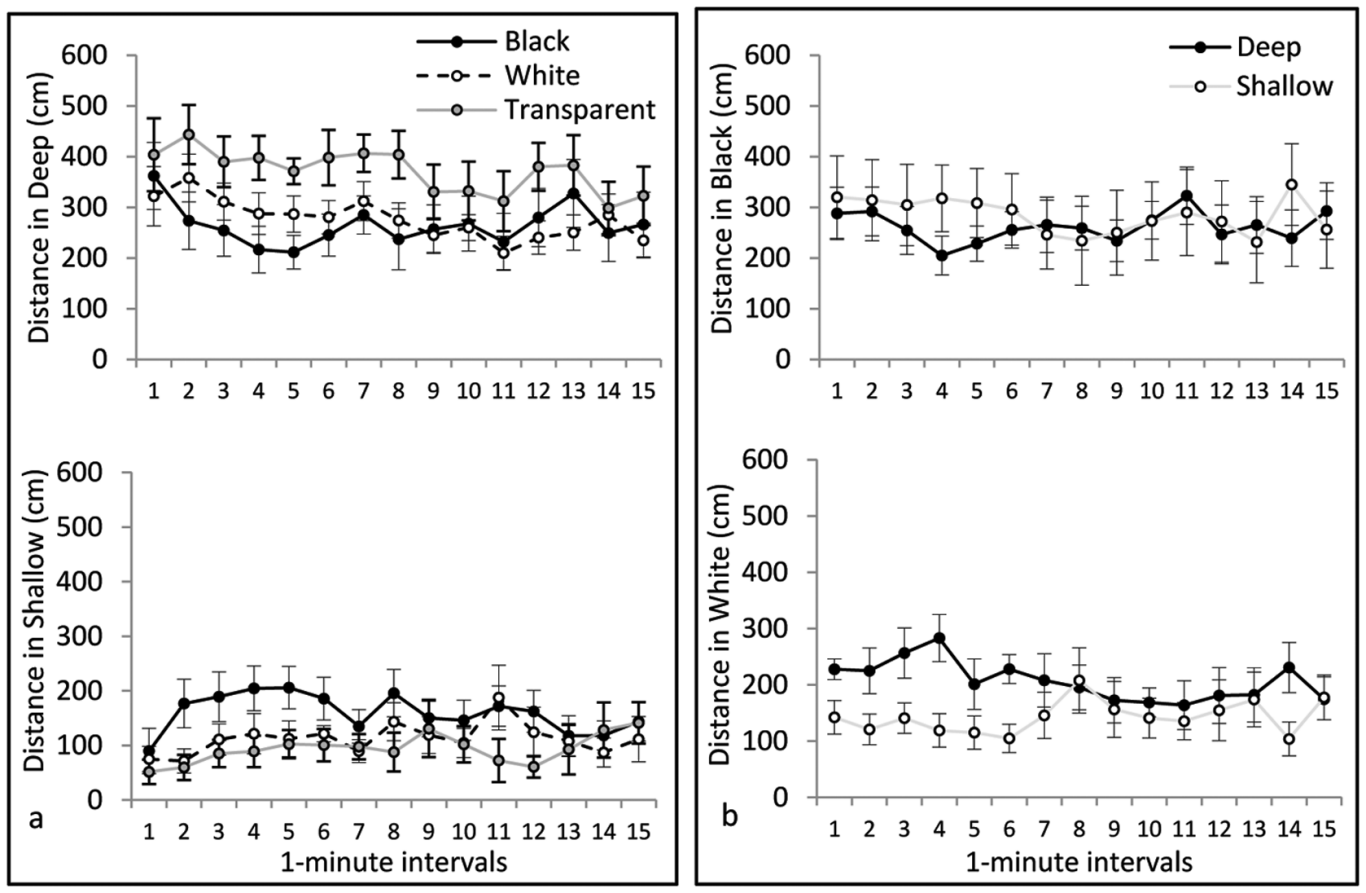
Path length. In the upper portion of **panel a** is the effect of black, white, and transparent stimuli on swim distance in the deep half of the tank, while in the lower portion of panel a is swim distance in the shallow half of the tank. In the upper portion of **panel b** is the effect of deep or shallow stimuli on swim distance in the black half of the tank, while in the lower portion of panel b is swim distance in the white half of the tank.

Although behaviors in the novel tank and black/white tests have not yet been examined from this perspective, there is considerable evidence suggesting that the two tests may not be interchangeable. Pharmacological studies, for example, have reported that some drugs affect behavior in both tests (such as acute exposure to Diazepam and Buspirone, and chronic exposure to Fluoxetine) [14], [19], [20] while other drugs affect only one of the measures, or produce mixed results (such as Chlordiazepoxide, Nicotine, Desipramine and Citaprolam) [19], [20], [21]. The validity of the novel tank test as a conditional measure of fear has been called into question by studies that have failed to produce consistent effects of predator stimuli on diving behavior. The effects of alarm pheromone on diving are equivocal [5], [22], [23], [24], and predator exposure (using live, 3D model, or 2D animated predators) has consistently failed to produce any effects on diving [5], [11], [12], [15], although more recently an animated overhead image was shown to produce a diving response [25]. Of course it is possible that these equivocal results reflect an inadequacy of the stimuli to induce fear in laboratory-reared fish, rather than of the test to measure it. It is so far unknown how these stimuli affect behavior in the black/white test, and therefore unclear whether the response to predator cues represents a divergence between the tests.

Although not explicitly tested, results of previous studies suggest that the two measures do differ on some of the predictions above. For example, in the black/white test, little habituation has been observed to white even after repeated exposures over multiple days [8]. The diving response, in contrast, has shown substantial habituation both within and between sessions, often within a few minutes [22]. Additionally, the diving response appears to exhibit a higher degree of variability than black/white preference, although a systematic comparison has not been undertaken. Finally, one previous study has indicated that confinement to a black, white, or transparent environment can produce effects on behavior in the novel tank test [3]. Taken together, these results suggest that depth stimuli may be ‘conditional’ – that is, fish only avoid the shallow part of a tank under certain motivational states – while black/white stimuli may be ‘causal’ – that is, the white compartment may actually be aversive to zebrafish. Further evidence for this possibility would be provided by information on the effects of black/white stimuli on diving, and the effects of depth stimuli on black/white preference. It has so far been impossible to directly examine the effects of black or white stimuli on diving, because the use of opaque black or white walls prevents recording vertical behavior. In the current study, an apparatus with two chambers differing in depth circumvents this problem, by allowing depth preference to be recorded from above the tank (as a function of side preference), obtaining a measure analogous to the black/white test [26]. Thus, the main goal of the current study was to symmetrically evaluate the effects of tank color on depth preference and of tank depth on color preference in zebrafish.

## Methods

### Subjects

Subjects were 59 adult wild-type (AB) zebrafish, of mixed gender, raised in the laboratory from a line originally obtained from the University of Oregon breeding facility. Subjects were housed in an Aquaneering table-top housing rack, with a recirculating filtration system using mechanical, biological, and chemical filtration. The subjects were housed in groups of 20, in 10 L system tanks. Because each subject was run in a single session, individuals were removed from the group of naïve fish, and then returned to a separate, identical tank containing experienced fish. The temperature of the tanks was held at 25°C, and the room was maintained on a 14/10 light/dark cycle. Subjects were fed 1–2 times daily on a mixed diet of live brine shrimp, freeze-dried brine shrimp, and Tetra-Min® flake food. The housing conditions and protocols were approved by the University of San Diego IACUC.

### Apparatus

The split-depth tank was a rectangular glass aquarium (20×15×20 cm; length×width×depth), like that described in Blaser & Goldsteinholm [26], [27]. In the ‘split’ configuration, one side of the tank was set to a depth of 10 cm using a plexiglas partition, and the other side set to a depth of 15 cm. In the ‘shallow’ configuration, both sides of the tank were set to a depth of 5 cm. In the ‘deep’ configuration, both sides of the tank were set to a depth of 15 cm. In all cases, gravel substrate was placed on a floor 5 cm below the plexiglas partition on each side. The sides of the tank were either left uncovered (transparent), covered in black paper (black), covered in white paper (white), or covered in black on one side, and white on the other. Figure 1 illustrates the apparatus for each configuration tested. The experimental setup was lit from above such that the inside of the apparatus ranged from 400 lux (in the black condition) to 600 lux (in the transparent condition). A video camera located approximately 1 m above the tank was used to monitor the location and activity of the fish. The video fed directly to a desktop computer which used Noldus Ethovision® to track the swim-patterns of the fish. The video-tracking data were then used to determine relevant measures of behavior including location in the tank (duration in each side, distance to the outer walls) and locomotor behavior (path length, immobility, shuttling).

### Procedure

#### Experimental Design

The animals were divided into five groups of 11–13 fish. Three of the groups (Depth Preference Groups – DP) were tested for depth preference while being confined to a single wall color (Black, White, or Transparent). All of these animals were tested in the split-tank configuration, with the walls either uncovered (transparent), or completely covered in black or in white. The other two groups (Color Preference Groups – CP) were tested for color preference while being confined to a single depth (Shallow or Deep). All of these animals were tested in a black/white tank, with the depth set to either 5 cm (Shallow), or 15 cm (Deep). Each subject was observed individually in a single session lasting 15 minutes. Subjects were gently netted from their home tank and placed into the center of the experimental tank. Recording began immediately, and continued for the entire 15 minutes of the test. After the test was complete, animals were returned to a separate home tank housing experienced individuals.

### Behavioral Measures

For the DP groups, the primary dependent measure was *Duration in Shallow*, which was defined as the duration of each 1-minute interval that the animal spent on the shallow side of the tank in seconds. For the CP groups, the primary dependent measure was *Duration in White*, which was defined as the duration of each 1-minute interval that the animal spent on the white side of the tank in seconds. Additional dependent measures included the *Distance from Walls* (average distance of the animal from the nearest outer wall), *Shuttling* (total number of center-crosses in each minute), *Path Length* (total swim path length of the subject in each 1-min interval), and *Immobility* (movement rate of <1 cm/sec).

### Statistical Analysis

Separate analyses were used for the CP and DP groups. For the CP groups, behaviors were analyzed using a 2×2×15 (Depth: Deep, Shallow×Side: Black, White×1-min Interval) repeated-measures Analysis of Variance (ANOVA), with Depth as a between-subjects measure and Side and Interval as within-subjects measures. For the DP groups, behaviors were analyzed using a 3×2×15 (Color: Black, White, Transparent×Side: Deep, Shallow×1-min Interval) repeated-measures ANOVA, with Color as a between-subjects measure and Side and Interval as within-subjects measures. The duration in shallow/white and shuttling behaviors were analyzed using only data from the less-preferred side, since the scores for one side are not statistically independent of the scores for the other side. A single-sample t-test was used to confirm that DP animals exhibited a significant preference for the deep side, and that CP animals exhibited a significant preference for the black side. Tukey's HSD was used for posts-hoc analysis as needed.

## Results

### DP Groups

*Duration in Shallow* can be observed in Figure 2a. Single-sample t-test indicated a significant overall avoidance of the shallow side (<50%; t(32) = −8.0, p<0.001). Repeated-measures ANOVA yielded a significant effect of Color (F(2, 30) = 7.56, p = 0.002), with post-hoc analysis indicating that animals in the black condition spent significantly more time in the shallow side of the tank than those in the transparent condition (p<0.05). Animals in the white condition did not differ significantly from either black or transparent. ANOVA yielded no significant effects of Interval on duration in shallow. Follow up single-samples t-tests, corrected for multiple comparisons (α = 0.016), indicated that animals in the black group showed no significant side preference (t(10) = −2.18, p>0.05), while animals in both the white group and the transparent group significantly preferred the deeper side (white: t (10) = −9.01, p<0.001; transparent: t (10) = −10.922, p<0.001). *Shuttling* is illustrated in Figure 3a. Repeated-measures ANOVA yielded a significant effect of Color (F(2, 30) = 5.67, p = 0.008), with animals in the transparent condition shuttling significantly less frequently than those in the black and white conditions (p<0.05). There were no significant effects of Interval on shuttling.

**Figure 5:**
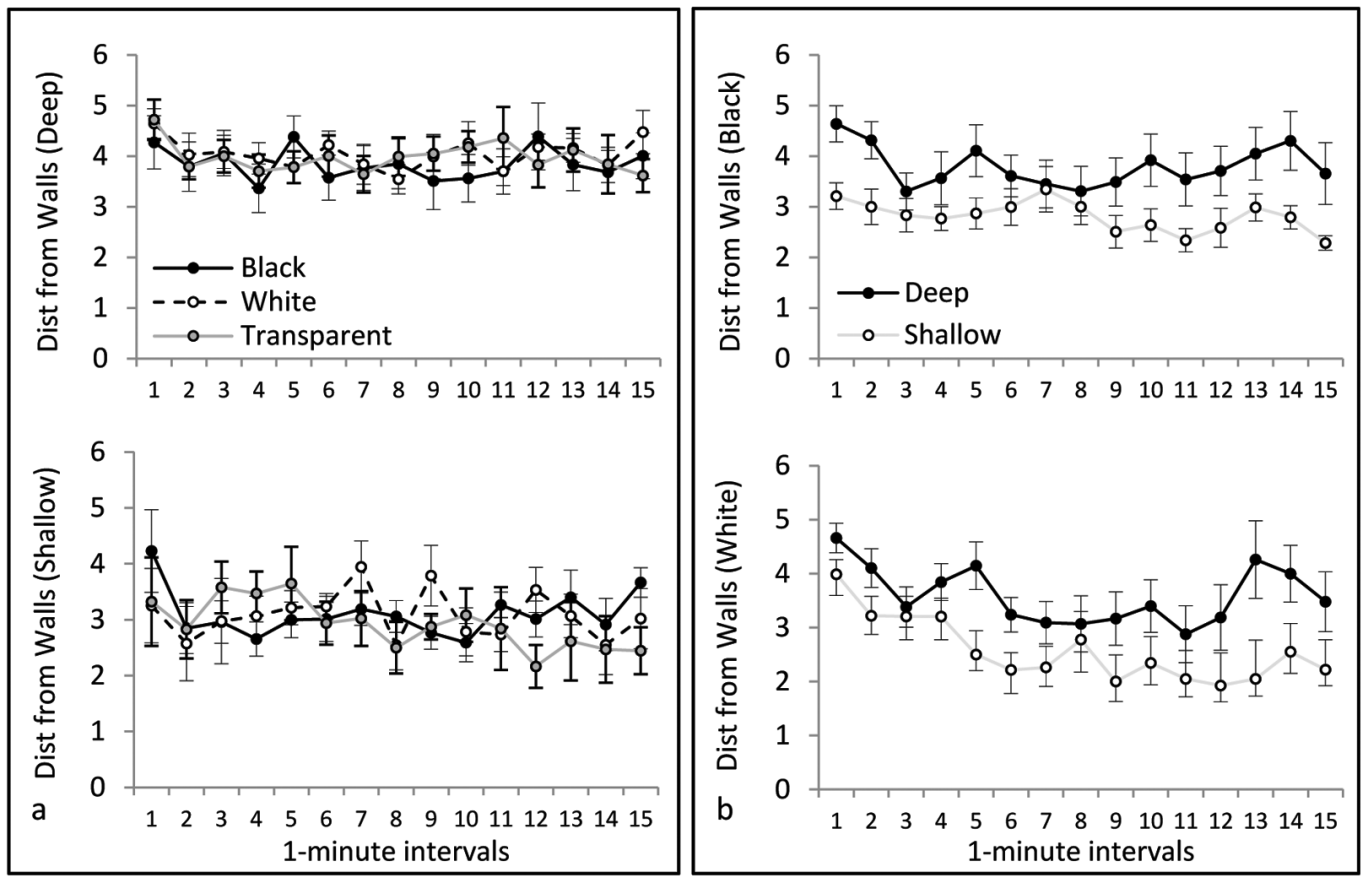
Mean distance from the walls. In the upper portion of **panel a** is the mean distance from the walls in the deep side of the tank, and in the lower portion of panel a is the mean distance from the walls in the shallow side of the tank. Black, white and transparent stimuli had no effect on distance from the walls. In the upper portion of **panel b** is the mean distance from the walls in the black side of the tank, and in the lower portion of panel b is the mean distance from the walls in the white side of the tank. Animals remained closer to the walls when the tank was shallow.

**Figure 6:**
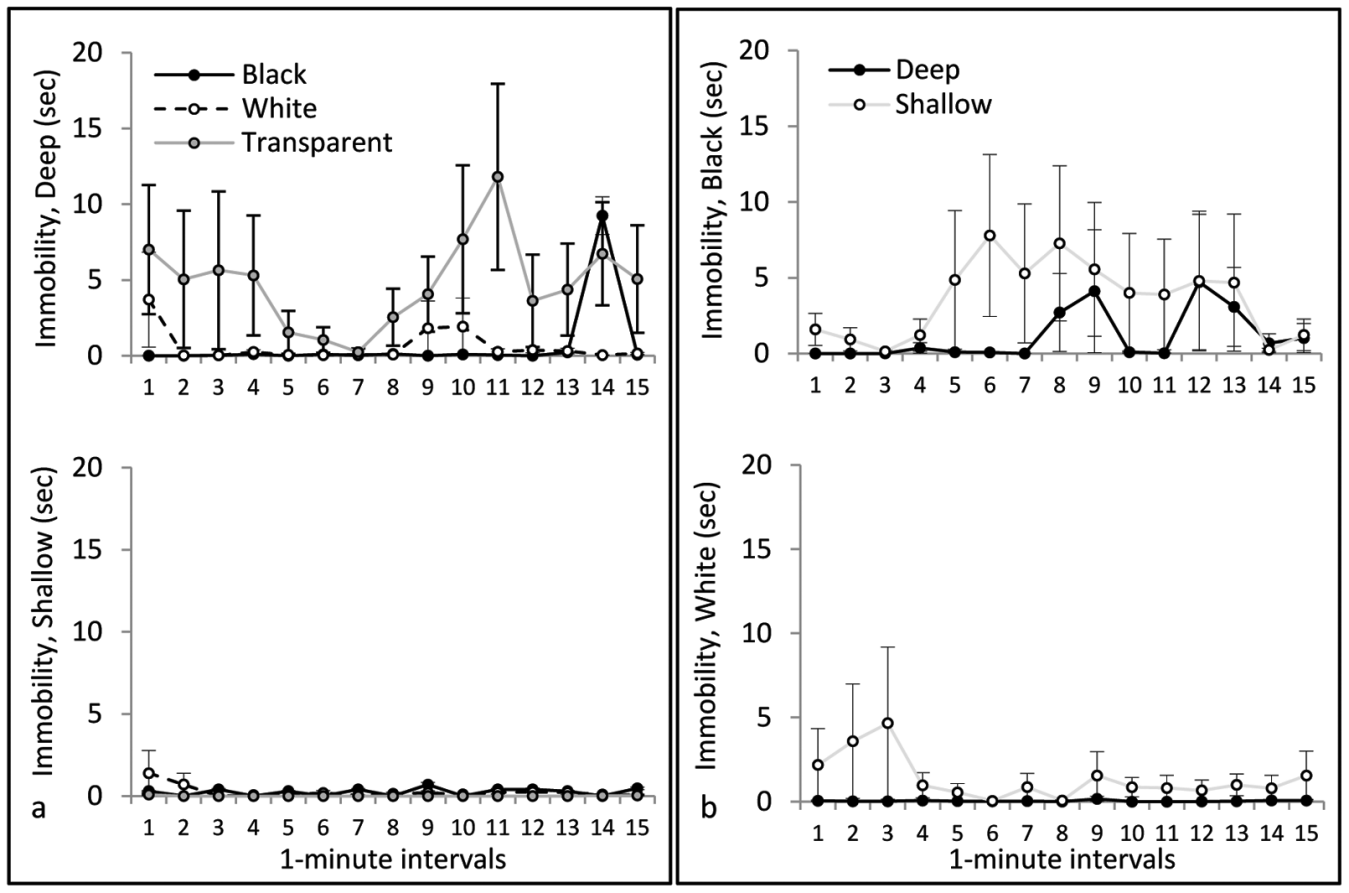
Immobility. In the upper portion of **panel a** is immobility in the deep side of the tank, and in the lower portion of panel a is immobility in the shallow side. Transparent stimuli produced more immobility than black or white stimuli, all of which was in the deep side. In the upper portion of **panel b** is immobility in the black side of the tank, and in the lower portion of panel b is immobility in the white side; there was no effect of depth on immobility.

*Path length* is illustrated in Figure 4a. Repeated-measures ANOVA yielded no significant main effect of Color on path length, indicating that confinement to black, white, or transparent tanks did not affect general locomotor activity levels. There was a significant difference between Sides (F(1, 30) = 63.11, p<0.001), with a greater swim distance in the deep side than in the shallow side, and a significant Color×Side interaction (F(2, 30) = 5.46, p = 0.009). This significant interaction reflects the fact that black-confined animals swam a greater distance in the shallow side than did white- or transparent-confined animals. There was a significant main effect of Interval (F(14,420) = 2.67, p = 0.001), with overall locomotor activity decreasing across the duration of the trial, and a Side×Interval interaction (F(14, 420) = 1.88, p = 0.03), with distance in the shallow side increasing across the duration of the trial. *Distance from Walls* is illustrated in Figure 5a. Repeated-measures ANOVA yielded no significant main effect of Color on this measure, indicating that confinement to black, white, or transparent tanks did not affect thigmotaxis in general. There was a significant difference between Sides (F(1, 30) = 30.22, p<0.001), with animals staying significantly closer to the walls in the shallow side than in the deep side, but there was no Color×Side interaction. No significant effects of Interval on thigmotaxis were detected. *Immobility* is illustrated in Figure 6a. Repeated-measures ANOVA yielded a significant effect of Color on immobility (F(2, 30) = 3.48, p = 0.044), produced by significantly more immobility in the transparent group than in the black group (p<0.05). There was also a significant difference between Sides (F(1, 30) = 5.66, p = 0.024), with more immobility in the deep side than the shallow side, and a significant Color×Side interaction (F(2, 30) = 3.85, p = 0.032). This significant interaction is because animals in the transparent group produced the most immobility, which was nearly all in the deep side. We did not find any significant effects of Interval on immobility.

### CP Groups

*Duration in White* can be observed in Figure 2b. Single-sample t-tests indicated a significant overall avoidance of the white side (<50%; t(25) = −2.26, p = 0.033). Repeated-measures ANOVA yielded no significant effect of Depth or Interval on duration in white. *Shuttling* is illustrated in Figure 3b. Repeated-measures ANOVA yielded a significant effect of Depth on shuttling (F(1, 24) = 10.00, p = 0.004), with animals in the deep condition shuttling significantly more frequently than those in the shallow condition. There were no significant effects of Interval on shuttling.

*Path length* is illustrated in Figure 4b. Repeated-measures ANOVA indicated no significant effect of Depth on path length, indicating that confinement to deep or shallow tanks did not affect general locomotor activity levels. There was no significant difference between the sides, and no significant Depth×Side interaction. There was a significant main effect of Interval (F(14, 336) = 1.90, p = 0.026), with locomotor activity decreasing across intervals, but there were no interactions between Interval and any other variable. *Distance from Walls* is illustrated in Figure 5b. Repeated-measures ANOVA indicated a significant effect of Depth (F(1, 24) = 5.28, p = .031), with animals in the shallow condition remaining significantly closer to the walls than those in the deep condition. There were also significant effects of Side (F(1, 24) = 13.98, p = 0.001), Interval (F(14, 336) = 4.83, p<0.001), and a Side×Interval interaction (F(14, 336) = 1.94, p = 0.22). On average, the mean distance from the walls decreased over time (thigmotaxis increased), and this change was greater in the white side than in the black side. *Immobility* is illustrated in Figure 6b. Repeated-measures ANOVA yielded no significant effects of Depth or Side on immobility.

## Discussion

**Figure 7:**
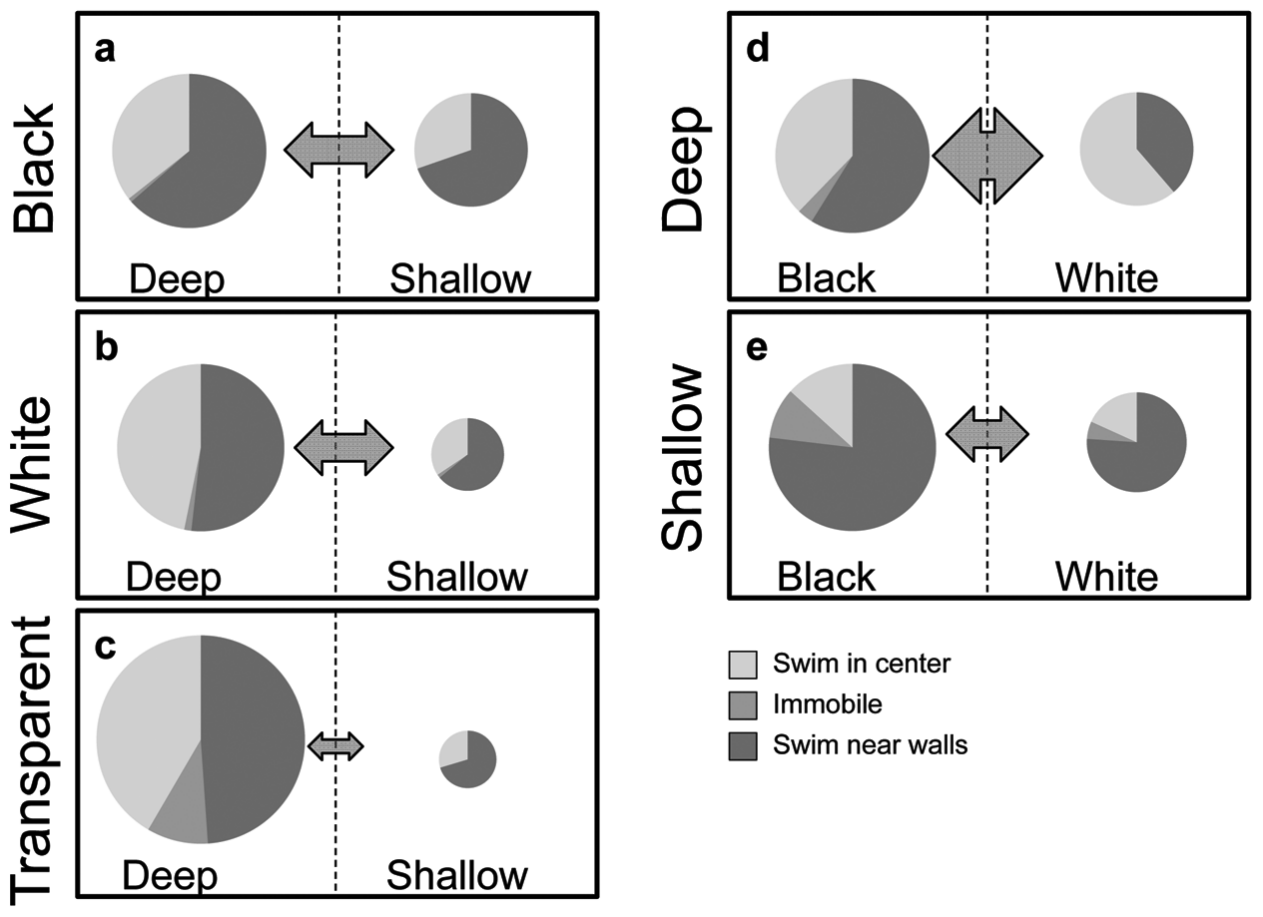
Schematic representation of behavior. In **panels a–c** are the DP groups, and **panels d and e** are the CP groups. Circle size represents the duration of time in each side, and arrow size represents the frequency of shuttling between the two sides.

The results observed in the current report provide further support for a putative dissociation between the effects of color and depth stimuli on zebrafish behavior. As predicted based on previous reports, animals given a choice between black and white sides of a novel tank preferred the black side, and animals given a choice between deep and shallow sides of a novel tank preferred the deep side. Manipulations of color stimuli significantly affected depth preference; when the walls were black, there was a reduction in preference for the deeper side. Conversely, manipulations of depth stimuli had little effect on color preference; animals showed similar avoidance of white whether confined to deep or shallow conditions. Additionally, manipulation of color affected shuttling and immobility, but not thigmotaxis; manipulation of depth affected shuttling and thigmotaxis, but not immobility. Figure 7 illustrates the general pattern of effects across all conditions.

The DP groups were confined to black, white, or transparent-walled tanks, and tested for depth preference. Animals in the black tanks exhibited the weakest preference for the deeper side (no significant side preference), while animals in the transparent tanks exhibited the strongest preference. In all groups, distance in the shallow side increased significantly over the first few minutes of the test, indicating rapid habituation (although a preference for the deeper side remained throughout the trial). These results are consistent with earlier reports that zebrafish in a traditional novel tank test gradually spend more time in the top portion of a tank over the first 6 minutes of the test [3], [14], [22]. Animals tested in the transparent tanks also exhibited more immobility, and less frequent shuttling behavior, than those tested in black tanks. Taken together, these results suggest that the transparent tank may induce more anxiety than either of the two opaque tanks, of which the black tank induced the least. These results are surprising, given that Blaser and Peñalosa [28] found little preference for a black chamber over a transparent chamber, but a strong preference for both transparent and black over white. They are also surprising in light of Rosemberg et al. [3], who found that confinement to black and transparent conditions significantly increased bottom-dwelling relative to white conditions.

One possible source of this discrepancy is the fact that exposure to the color stimuli in Rosemberg et al. [3] happened prior to measuring the diving response, while measurement here was concurrent. It is possible that in the previous study as in this one, white confinement produced a stronger diving response than black confinement, which could have therefore begun to habituate. In the subsequent test, this habituation may have generalized to produce less diving following white exposure than black exposure. It is unclear why behavior in the transparent tank in both Rosemberg et al. [3] and Blaser and Peñalosa [28] should resemble the black tank over the white tank, while in the current study it more closely resembles the white tank. Although the lighting conditions here are similar to those used in the Blaser and Peñalosa study, it is possible that the brighter lighting in the transparent condition relative to black or white could have produced the effect [29]. Perhaps an important message here is that ‘transparent’ is not a unitary stimulus, but rather a condition which allows visual stimulation to vary depending on the ambient laboratory conditions. The degree of fear or exploration exhibited in ‘transparent’ conditions may depend more on the presence of experimenters or other movement in the laboratory, objects placed near the tank, and other extraneous stimuli, than on the tank material itself. It is possible, therefore, that recording behavior from above an opaque tank will produce more reliable results across laboratories than recording behavior from a transparent tank, despite the limitations of this approach.

The CP groups were confined to shallow or deep tanks, and tested for color preference. Animals in both groups exhibited a similar preference for the black side over the white side, and consistently with previous results [8], [30], no evidence of habituation in black preference was observed. Those tested in the shallow tanks exhibited less shuttling behavior, and more thigmotaxis, than those tested in the deep tanks. Depth did not affect immobility or path length. Combined with the results from the DP groups, it appears that forced exposure to color/luminosity stimuli affects depth preference, but forced exposure to depth stimuli does not affect color preference; when differences in behavioral measures are included, the evidence suggests that the tests measure dissociable mechanisms of behavior. Our data support the possibility that the black/white stimuli exert ‘causal’ influence on anxiety: forced exposure to these stimuli affects other measures of anxiety such as diving and immobility, and the preference does not readily habituate. In contrast, depth stimuli may exert ‘conditional’ influence: forced exposure to depth did not affect color preference or immobility, and depth preference, which may be initially produced by handling stress or novelty, habituates rapidly.

This behavioral dissociation between the novel tank and the black/white tests is consistent with pharmacological evidence from anxiolytic and anxiogenic drugs described in the literature [1], [14], [21], [22], as well as previous behavioral results [3]. It is possible that, as suggested by Ramos et al. for rodents [10], the behavioral repertoire of zebrafish in each test involves the recruitment of different genes, metabolic pathways or even protein expression, although the physiological mechanisms will require further investigation. The only common effect on behavior of the two stimulus types was shuttling; there was more shuttling in black than in white or transparent, and more shuttling in deep than in shallow. Because shuttling was not closely related to either immobility or path length, it is clear that shuttling is not simply an analogue of locomotor activity. It seems likely that shuttling in the two tasks reflects different processes; for example, in the DP groups, shuttling requires transitioning into the upper portion of the tank, and therefore may be related to vertical exploration (the higher frequency of shuttling in black is consistent with the higher proportion of time spent in the shallow side). In the CP groups, on the other hand, it might reflect different locomotor patterns in the deep and shallow conditions. Due to the increase in thigmotaxis in the shallow condition (often produced by thrashing or escape behaviors along the side walls and corners), behavior directed toward the side walls may have competed with the tendency to cross through the tank center.

Further characterization of the convergence or divergence of these tests would be useful to better understand the constructs being measured by each. One approach would be to concurrently compare the effects of variables such as stress, drugs, or genetic manipulations on both tests. Because only the AB strain was used here, it is possible that different populations or genetic strains of zebrafish, which have already been shown to differ on exploratory variables in previous studies [14], [21], [31], would exhibit different patterns of behavior than those reported here. Developing a single apparatus containing both types of stimuli may increase the reliability, efficiency (both in speed of experimentation and a reduced number of animals), and comprehensiveness of the test for large-scale screening. On a practical note, our results suggest that it may be unwise to make general claims about a drug or genotype based on behavior in just one of these tests, until both are more clearly understood. Both are appealing due to a high degree of face validity and efficiency of testing, but further research investigating the differences between motivational mechanisms in these tests will be useful for interpreting the effects of pharmacological or genetic manipulations on behavior [32]. Continued examination will ultimately provide sufficient evidence for construct and predictive validity, the most important types of validity for making generalizations to human behavior.
